# Mr. Gladstone on Medicine

**Published:** 1894-05-12

**Authors:** 


### The Hospital

An Institutional Journal of

The Medical Sciences and Hospital Administration, with

Vol. XVI.—No. 398.] AN EXTRA NURSING SUPPLEMENT. [May 12, 1894.

### Mr. Gladstone on Medicine.

Mr. Gladstone has often publicly professed a great regard for medicine and a profound conviction of its importance to civilised mankind. To civilised and highly differentiated mankind in especial must physiological knowledge and the capacity for preserving health under difficulties be a subject of constantly increasing importance. "While wealth increases," said Mr. Gladstone, at Prince's Hall, when speaking on behalf of the proposed memorial to the late Sir Andrew Clark, "while wealth increases, while inventions and discoveries increase, wants will increase and enjoyments will increase, and in connection with wants and enjoyments there will, I fear, be a corresponding development of infirmity and disease. The medical profession braces itself to grapple with the situation which has been created, and continually advances, in knowledge, credit, and importance." The obvious truth is that if wants and enjoyments increase there must also be readily available the knowledge which is able to choose discriminatingly what wants are legitimate, and also what enjoyments are advantageous and safe, and what are dangerous and injurious. For it would be a poor result of culture and material progress if the more educated classes of the community should become too feeble in body and mind for duty, and enjoyment, and for those conflicts of life which, as they become higher and nobler, demand more strenuousness and staying power both physical and mental. It is absolutely imperative, therefore, in a great, cultured, and progressive state like our own that there should be an order of scientific, practically capable, and trustworthy medical thinkers and teachers, whose guidance shall be always and everywhere available for all classes of the community.

Not seldom when a man sees himself as others see him he experiences sensations which are the reverse of agreeable. But this is by no means the case with the medical profession when it looks at itself with Mr. Gladstone's eyes. We do not wish to take too literally all the kind things the ex-Premier says of the medicine of modern times. But it would be an advantage to the community, and, we think, nothing more than a just reparation to medicine, if the educated public generally would borrow Mr. Gladstone's spectacles when it sits in judgment upon us. There are those who do not give medicine the credit which it deserves for scientific attainments which are undoubtedly high, for practical capacity to ameliorate human ills which is exceedingly great, for professional morals which are not only severe but austere, and for a spirit which is resolute for improvement almost beyond parallel. These marks of modern medicine are very obvious to Mr. Gladstone, as we know they also are to the Marquis of Salisbury, and to all other educated persons who can see with the eyes of the philosophical observer. "Great," says Mr. Gladstone, "as has been the position of the medical profession in our generation, and in some generations previous, it is a position continually advancing and continually widening. . . The position of the profession at the present day has become one of vital and commanding interest to the whole of society; and I anticipate that that interest must continue."

It is often wondered in these days of stress and strain whether, after all, "the game is worth the candle"; whether, in spite of our most strenuous and exhausting labours, the world makes any forward movement which is worthy of the name of progress. Reactionaries point us to Homer, to Egyptian sanitation, and to the astonishing dramatic power of the book of Job. To all these reactionary questionings the solid and massive gains in physiological knowledge, and the wonderful developments in practical medicine and surgery of the present century furnish answers absolutely final. For good or for ill, the real knowledge of man and of all things which have life and movement, or which can be seen by the eye and touched by the finger, is penetrating rapidly into the common mind; and never again, unless the whole of civilisation should be submerged in a Noachian deluge, can there be any other world for us to live and work in but a world which is charged with accumulated knowledge of great and small, of near and far, of things seen and things unseen, of mysteries in earth and sea, in sun and star, which affright the imagination whilst they stimulate and expand the mind. Much of this knowledge has been conquered and is held captive by medicine. More knowledge still, infinitely more, is waiting to be conquered; and that very spirit which has raised Britain from a Heptarchy to an Empire, and planted her flag upon every strand, animates the modern soldiers of physic, and will never permit them to sheathe the sword of scientific progress until all which may be known by mortal man is conquered and made serviceable for human needs.

A note of warning Mr. Gladstone gives us; and we take it from a Chrysostom so much greater than the eloquent teacher of the primitive church, with the reverence which venerable age and unparalleled service to country are so fitted to excite in our minds. Knowledge, says he, is not of itself sufficient. It is not sufficient for any profession or calling, least of all, perhaps, for the members of the profession of medicine. "In every profession," to quote Mr. Gladstone's own words, "there is something more, I would almost say more important, required than knowledge and capacity, and that is character. The position of medical men is one which brings them more and more within the intimacy of the family circle, which brings them more and more into the confidence of those for whose benefit they are consulted; and in order that they may be worthy of that confidence and turn it to account it is not requisite merely that they should have studied with fidelity and even with ardour the difficult subjects to which they devote their minds. But it is requisite that all the qualities which give force and weight to character should be exhibited by them in conjunction with their professional skill." The leaders of medicine have long been convinced of the soundness of these principles, and so thoroughly do such views accord with those of the general public on the question that it is fast becoming, not merely difficult but impossible, for any man who is not a man of real principle to gain and retain a foothold in the medical profession. It does not seem to have struck the public that our attitude on such a burning question as that, for example, of professional advertising is one which not only conserves the morals of our own profession, but one which indirectly must influence all classes of advertisers in a most marked degree. The medical man must not advertise at all, we say, because if he advertises that only which is true, he cannot affirm that he is either cleverer or more successful than hundreds of his brethren; and to advertise that which is not true would be to degrade both himself and his brethren. Therefore, let his works and his character alone speak for him. But when this attitude of mind is taken up by the most scientific and one of the most cultured of all the professions, it cannot but exercise a very potent influence on all classes of the public who can observe and think.

The medical profession, we think, owes some gratitude to men like Mr. Gladstone and the Marquis of Salisbury for recognising and pointing out to the public the obvious fact that it has long been casting, and has now finally cast its unscientific slough, and stands before the world a protagonist of observation, of experience, and of pure reason. The public, too, should be grateful, and should be more ready to recognise the fact, which has long been so obvious to those who have looked with the eyes of the philosopher. Medicine now stands for actual knowledge, for the serviceable knowledge of man. The public interest demands that every professor of the medical art shall be charged to the full with snch knowledge, and shall be of such character that his knowledge will be used not for himself but only for the public well-being. Let the public try to discover the large obligations it now owes to a regenerated and scientific medicine. When that has been done, it will have taken the wisest of all steps towards keeping aflame that candle of pure medical science which, once having been effectually lighted, ought never more to be put out.

